# Correction to ‘A single bout of prior resistance exercise attenuates muscle atrophy and declines in myofibrillar protein synthesis during bed‐rest in older men’

**DOI:** 10.1113/JP289816

**Published:** 2025-08-20

Smeuninx, B., Elhassan, Y.S., Sapey, E., Rushton, A.B., Morgan, P.T., Korzepa, M., Belfield, A.E., Philp, A., Brook, M.S., Gharahdaghi, N., Wilkinson, D., Smith, K., Atherton, P.J., Breen, L. (2025). A single bout of prior resistance exercise attenuates muscle atrophy and declines in myofibrillar protein synthesis during bed‐rest in older men. *The Journal of Physiology*, 603(1), 87–105. https://doi.org/10.1113/JP285130

This article corrects the affiliations listed for Paul T. Morgan. A second affiliation should have been included in the original article, as below:

Department of Sport and Exercise Sciences, Institute of Sport, Manchester Metropolitan University, 99 Oxford Road, Manchester, UK

The authors apologise for this error.

